# Ultra-High-Risk that do not transition to psychosis. What happens?

**DOI:** 10.1192/j.eurpsy.2024.1533

**Published:** 2024-08-27

**Authors:** M. B. Ruas Resende, F. Agostinho, R. Nogueira, D. Cotovio, F. A. Silva, R. Lousada

**Affiliations:** Psychiatry, Hospital Beatriz Ângelo, Lisbon, Portugal

## Abstract

**Introduction:**

Speaking prospectively we use the concept of “at risk mental state” (ARMS) to describe the state in which a person has a heightened risk of developing a psychotic disorder. Young people who are experiencing ARMS can be more precisely defined as being at ultra-high-risk of psychosis using a specific set of criteria known as the UHR criteria.

**Objectives:**

To clarify the concept of ultra-high-risk individuals and to characterize the clinical and functional characteristics and general psychopathology of those individuals that do not transition to psychosis during the follow-up period.

**Methods:**

Research on UpToDate using the terms “Ultra-High-Risk”; “psychosis”, “transition”.

**Results:**

Recent literature has suggested that less than 30% of those who meet established criteria for being at Clinical-High-Risk of psychosis (CHR-P) go on to develop a psychotic illness. It is therefore of crucial importance and relevance to assess and clarify what happens to high-risk individuals who do not transition to psychosis, who make up the vast majority.

One of the most recent studies (NAPLS-2) that encompassed 764 of CHR-P individuals who were followed for 2 years, concluded that 278 did not transition to psychosis during the follow-up period. Three clinical outcomes were recorded: 1 group had experienced a psychopathological remission (39.57%); the other kept symptomatic but not currently meeting criteria for a prodromal risk syndrome (33.45%); the third group had a prodromal progression (26.98%). The study concluded among others that although the remission group had improved social functioning at 2 years compared with the other groups, they were still functioning below the healthy control group.

Another meta-analysis that included a total of 2756 CHR-P individuals with a mean duration of follow-up of 30.7 months evaluated several clinical outcomes in CHR-P that didn’t transitioned to psychosis and between CHR-P non-transitioning versus those transitioning to psychosis. It concluded that CHR-P that do not transition to psychosis have an overall improvement of symptoms (APS, negative, depressive) and functioning at follow-up compared to baseline.

**Conclusions:**

The occurrence of a first psychotic episode is often devastating for the patient and their family, especially given its usual onset in adolescence and early adulthood. This is a critical period in the individual’s development as a person, and disorders at this stage can threaten the potential for a productive and inclusive adult life. Studies have suggested that less than 30% of individuals classified as UHR actually develop a psychotic disorder.

However, little is known about the individuals belonging to this group who do not transition to psychosis. We therefore consider it is relevant to clarify the clinical and functional outcomes of this group of individuals.

**Disclosure of Interest:**

None Declared

